# Dihydroartemisinin inhibits metastatic potential and cancer stemness by modulating the miR-200b–BMI-1/VEGF-A axis in ovarian cancer

**DOI:** 10.1038/s12276-025-01582-2

**Published:** 2025-12-05

**Authors:** Jin Gu Cho, Sung Wook Kim, Eunsik Yun, Sumin Yoon, Jihea Choi, Dasol Yeom, Aram Lee, Dawn Lee, Su Jin Jeong, Woochul Chang, Woo Yeon Hwang, Youngsun Kim, Kiyong Na, Ki Hyung Kim, Dong Soo Suh, Kyung Un Choi, Jong Hoon Park, Keun Il Kim, Kyung Hyun Yoo, Byung Su Kwon, Jongmin Kim

**Affiliations:** 1 Division of Biological Sciences, Sookmyung Women’s University, Seoul, Republic of Korea; 2 Research Institute for Women’s Health, Sookmyung Women’s University, Seoul, Republic of Korea; 3 Department of Obstetrics and Gynecology, Kyung Hee University College of Medicine, Kyung Hee University Medical Center, Seoul, Republic of Korea; 4 Kyung Hee University Medical Center, Medical Science Research Institute, Statistics Support Part, Seoul, Republic of Korea; 5 Department of Biology Education, College of Education, Pusan National University, Busan, Republic of Korea; 6 Department of Pathology, Kyung Hee University Hospital, Kyung Hee University College of Medicine, Seoul, Republic of Korea; 7 Department of Obstetrics and Gynecology, Pusan National University School of Medicine, Biomedical Research Institute, Pusan National University Hospital, Pusan, Republic of Korea; 8 Department of Pathology, Pusan National University Hospital, Busan National University School of Medicine, Busan, Republic of Korea

**Keywords:** Cancer stem cells, Cancer stem cells

## Abstract

Despite therapeutic advances, ovarian cancer remains a major clinical challenge owing to its frequent metastasis and chemoresistance, which are often driven by cancer stem cells (CSCs) and proangiogenic signaling. Here we demonstrated that dihydroartemisinin (DHA), a derivative of the antimalarial drug artemisinin, inhibits CSC characteristics, tumor neovascularization and resistance to carboplatin via a microRNA-dependent mechanism in ovarian cancer. DHA substantially inhibited CSC properties, tumorigenicity and vascular endothelial growth factor A (VEGF-A)-mediated tumor neovascularization in ovarian cancer. Moreover, the combined treatment with DHA and carboplatin produced a synergistic effect that reduced tumor burden, chemoresistance and peritoneal dissemination in vivo. Mechanistically, DHA downregulated BMI-1 and VEGF-A/vascular endothelial growth factor receptor 2 (VEGFR2), which are critical factors in CSC maintenance and metastasis, via the upregulation of miR-200b. An analysis of ovarian tumor tissues collected from patients enrolled in our clinical cohort revealed that dual positivity for BMI-1 and VEGF-A was associated with poor progression-free survival. Overall, DHA targets the miR-200b–BMI-1/VEGF-A axis to suppress cancer stemness and metastatic potential, highlighting its therapeutic promise in overcoming the limitations of standard chemotherapy for ovarian cancer. The clinical trial number for this study is not applicable.

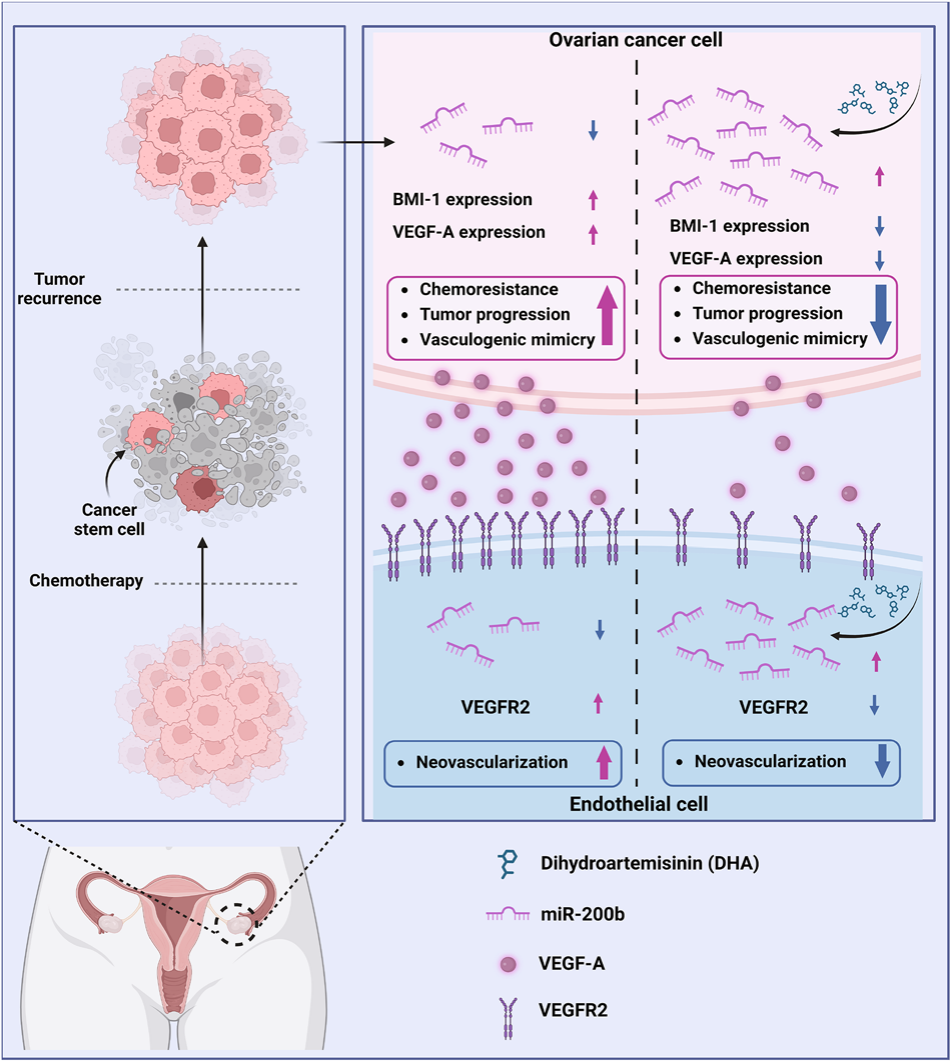

## DHA combats ovarian cancer stemness and metastasis

Ovarian cancer is a deadly disease, often treated with platinum-based drugs. Researchers explored a new approach using dihydroartemisinin (DHA), a compound derived from a traditional Chinese herb, to tackle this issue. The study focused on cancer stem cells, which are believed to cause cancer recurrence and resistance to treatment. The researchers tested DHA on ovarian cancer cells and found it reduced the ability of these cells to form spheroids and decreased their survival. They also discovered that DHA lowered the levels of BMI-1 and vascular endothelial growth factor A, proteins linked to cancer growth and blood vessel formation. In experiments with mice, DHA treatment led to smaller tumors and less spread of cancer within the abdomen. The study suggests that DHA could enhance the effectiveness of existing treatments by targeting cancer stem cells and reducing tumor growth.

This summary was initially drafted using artificial intelligence, then revised and fact-checked by the author.

## Introduction

Ovarian cancer (OC) is the most fatal of all gynecological cancers and ranks as the fifth leading cause of cancer-related deaths among women. The mortality rate of this disease has only slightly improved over the last 30 years^1–3^. Platinum-based antineoplastic drugs are commonly used to treat OC^4^. Although most patients with OC initially respond well to platinum-based chemotherapies, their long-term survival and cure rates remain low owing to acquired tumor resistance over time, which may subsequently lead to metastasis^5,6^. Therefore, targeted therapies have attracted considerable attention as promising alternative strategies. Several molecules, including agents targeting epidermal growth factor receptors (EGFRs), poly (ADP-ribose) polymerase (PARP) and WEE1 kinase, have been evaluated for use in OC^7–9^. Although most phase III trials of currently approved targeted therapies, such as PARP inhibitors, have reported improvements in progression-free survival (PFS), overall survival (OS) has not improved in selected high-risk cases^10^. Moreover, the cost–benefit ratios of these targeted therapies remains controversial^11^. Therefore, there is an urgent need to develop drugs with novel mechanisms of action to overcome drug resistance and prevent cancer relapse and metastasis.

The cancer stem cell (CSC) hypothesis suggests that heterogeneous tumors contain a small fraction of stem-like cancer cells with unique properties. Cancer recurrence is thought to be driven by the survival of a small percentage of CSCs that are capable of resisting chemotherapy, repopulating the tumor and metastasizing to distal sites^12–14^. BMI-1, a member of the polycomb group of proteins^15^, is highly enriched in CSCs and is associated with advanced invasive stages of tumor progression^16^. The ability of CSCs to trigger cancer recurrence after chemotherapy has been attributed to the activation of several proteins, including BMI-1^17,18^. BMI-1 functions as a stem cell factor in cancer initiation, progression and chemoresistance, making it a potential therapeutic target^19^. As an oncogene, BMI-1 allows cancer cells to evade apoptosis by modulating multiple growth signaling pathways^17^. BMI-1 is overexpressed in a high percentage (72.5%) of OC cases. Studies have indicated that abnormal expression of BMI-1 is important for acquiring an invasive and/or aggressive phenotype and is associated with poor prognosis and clinical outcomes in OCs^20^. Considering its role in chemoresistance and maintaining the self-renewal ability of CSCs, silencing *BMI-1* may promote sensitivity to chemotherapy and prevent cancer relapse and metastasis.

Angiogenesis, the formation of new blood vessels from preexisting vessels, is important for tumor survival and development^21^. Tumor cells can alter their metabolism and release proangiogenic factors to survive intratumoral hypoxia^22,23^. Vascular endothelial growth factor (VEGF) is essential for neovascularization in multiple cancers, including OC^24,25^. Under hypoxic conditions, hypoxia-induced factor-1α is upregulated and binds to the promoter region of the *VEGF* gene to stimulate angiogenesis^26^. Furthermore, VEGF upregulation contributes to increased microvessel density, metastasis and poor diagnosis^27^. VEGF is also overexpressed in recurrent and chemoresistant OCs^28^. Vasculogenic mimicry (VM), an alternative mechanism for tumor neovascularization, involves the creation of a blood supply system that provides oxygen and nutrients to inner tumors independently of angiogenesis, enabling proliferation and metastasis^29^. VM occurs in numerous aggressive tumors, including OC, glioma and breast cancer^30–32^, and CSCs in many tumor types directly participate in VM formation^33,34^. CSCs are a vital source of tumor neovascularization inducers, including VEGF, which drives angiogenesis and VM, and contribute to tumor progression and metastasis. Therefore, the dual blockade of CSC properties and tumor neovascularization could represent an effective therapeutic strategy for treating aggressive, metastatic and drug-resistant OCs.

Dihydroartemisinin (DHA)^35^, the most active derivative of artemisinin, is isolated from the traditional Chinese herb *Artemisia annua* L. DHA was approved by the US Food and Drug Administration in April 2009 for the treatment of malaria. DHA also exhibits selective anticancer effects against ovarian, pancreatic, hepatocellular and breast cancers^36,37^. Its anticancer effects are associated with multiple mechanisms, including reactive oxygen species generation, oxidative DNA damage induction, maintenance of DNA double-strand breaks, autophagy and apoptosis. Clinical trials on malaria therapy have shown that artemisinin derivatives are well tolerated with minimal side effects^38^, supporting the potential therapeutic application of DHA in oncology. However, the ability of DHA to target various components associated with ovarian CSCs and tumor neovascularization and the underlying mechanisms remain elusive.

Therefore, we aimed to investigate the effects of DHA on CSC traits, VM and angiogenesis while elucidating underlying molecular mechanisms. This study provides new evidence that DHA eradicates CSC characteristics and tumor neovascularization by regulating the miR-200b–BMI-1/VEGF-A/VEGFR2 axis, thereby improving outcomes in patients with platinum-resistant relapse, a major obstacle in the clinical management of OC.

## Materials and methods

### Cell culture and treatment

Human OC cell lines (SKOV3 and OVCAR3) were cultured in Roswell Park Memorial Institute 1640 medium (HyClone) supplemented with 10% fetal bovine serum (HyClone), 1% penicillin–streptomycin (WelGENE) and MycoZap (Lonza). Human umbilical vein endothelial cells (HUVECs) were cultured in EGM-2 medium (Lonza) supplemented with 1% penicillin–streptomycin. HUVECs obtained a least before the seventh passage were used for experiments. Both cell lines were maintained at 37 °C in a 5% CO_2_ incubator. A fresh ovarian tumor mass was placed in saline and kept on ice to maintain cell viability. The ovarian tumor tissue was minced with scissors and enzymatically digested with collagenase type I (Sigma-Aldrich) at 37 °C for 1 h. The primary OC cells were then collected by passing through a 0.22-μm sterile filter and suspended in Dulbecco’s modified Eagle medium. DHA (Sigma-Aldrich) and carboplatin (CBP; Sigma-Aldrich) were dissolved separately in dimethyl sulfoxide (DMSO). In all experiments, the drugs were administered at concentrations of 10 and 50 µM for 72 h.

### Preparation of human tissue specimens

The study protocol was approved by the Institutional Review Board of Pusan National University Hospital (IRB-2001-010-087). All patients provided signed informed consent before study initiation. The ovarian tumor tissue was collected during surgery and was verified by a pathologist from the Department of Gynecologic Pathology before transfer to the cell culture laboratory. Fresh, pathologically confirmed samples were processed using enzymatic and mechanical dissociation to obtain single-cell suspensions, which were cultured under conditions suitable for stem cell culture. The RNA and proteins were extracted from cultured tumor tissue.

### Spheroid-formation assay

The SKOV3 cell line was derived from the ascites fluid of a patient with highly metastatic OC. SKOV3 cells are relatively resistant to chemotherapy and have been shown to metastasize in a mouse model^39^. Therefore, we used a spheroid-formation assay to mimic metastatic conditions and enrich ovarian CSCs. SKOV3 and primary OC cells (1 × 10^5 ^cells per milliliter) were seeded in six-well plates coated with poly 2-hydroxylethyl methacrylate (Sigma-Aldrich). The cells were cultured in serum-free Dulbecco’s modified Eagle medium/F12 medium (Welgene) supplemented with 20 ng/ml epidermal growth factor (Invitrogen), 10 ng/ml basic fibroblast growth factor (Invitrogen), 0.4% bovine serum albumin (Gendepot) and 5 mg/ml insulin (Sigma-Aldrich). The cells were then treated with DHA or CBP. The spheroid formation was assessed 5 days after seeding. The cells were observed under a microscope, and the spheroid size was measured using ImageJ.

### Flow cytometry

The ALDEFLUOR Kit (Stem Cell Technologies) was used to detect aldehyde dehydrogenase (ALDH) activity. For the analysis of individual cells, spheroids were dissociated using cell dissociation buffer (Thermo Fisher Scientific), and the resulting suspended cells were centrifuged at 1000 rpm. Subsequently, the cells (1 × 10^6^) were resuspended in 1 ml of ALDEFLUOR assay buffer containing the ALDH substrate. The cells were incubated for 45 min at 37 °C before analysis. All assessments were performed using a FACS Canto II flow cytometer (BD Biosciences) at the Chronic and Metabolic Diseases Research Center, Sookmyung Women’s University, and the data were analyzed using FlowJo software.

### Housing of experimental animals

Female BALB/c nude mice (7–8 weeks old) were procured from Orient Bio and housed in a specific pathogen-free facility under a 12-h light and 12-h dark cycle at a humidity range of 40–60%. Ethical approval for animal experiments was obtained from the Animal Experiment Ethics Committee of Pusan National University Hospital (PNUH-2017-110), and the study adhered to the ethical guidelines of the institutional animal care and use committee regulations of Pusan National University Hospital.

### Subcutaneous tumor model

Female BALB/c nude mice (6-week-old, *n* = 10) were divided into two groups (DHA-treated and control, *n* = 5 per group). SKOV3 spheroids at a density range of 1.25 × 10^6^ to 1 × 10^7^ were injected into both flanks of each mouse (100 µl per injection). DHA (50 mg/kg) was administered twice weekly starting the day following transplantation. After 14 days, the tumors were collected, and their sizes were measured.

### Intraperitoneal tumor model

SKOV3 spheroids at a density of 5 × 10^6^ cells were intraperitoneally injected into each mouse (200 µl per injection) to establish a SKOV3 tumor model. At 3 days after injection, tumor-bearing mice were randomly divided into four groups (Control, CBP, DHA and CBP and DHA; *n* = 8 per group). From day 7 after injection, the mice in the single-treatment groups were intraperitoneally injected weekly with CBP (no. C2538, Sigma-Aldrich, 50 mg/kg body weight) or DHA. To mitigate the toxicity associated with CBP and DHA combination therapy, 40 mg/kg body weight CBP and 40 mg/kg body weight DHA were injected intraperitoneally once weekly. The body weights of the mice were measured every 2–3 days beginning on the day of injection. The mice were monitored weekly for ascites development and tumor growth. The tumor growth was measured using electronic calipers, and both the long and short axes were recorded. The mice were killed on day 28 post cell injection. Upon death, the abdomen of each mouse was dissected to measure ascitic fluid volumes exceeding 1 ml. In addition, images were obtained of tumors formed in the abdominal cavity, and their sizes were determined by isolating each tumor and weighing it on a microscale. The tumor samples were processed for histological analysis before fixation in 10% formalin.

### IHC

Immunohistochemistry (IHC) staining was performed on formalin-fixed paraffin-embedded tissues using a BMI-1 mouse monoclonal antibody (SC-390443; Santa Cruz Biotechnology). The counterstaining was performed using hematoxylin. The images were captured at 400× magnification using Aperio ImageScope software (Leica Biosystems).

### In vitro limiting dilution assay

The cells were seeded in poly(2-hydroxyethyl methacrylate)-coated 96-well plates at various concentrations (1–128 cells per well). DMSO and 50 µM DHA were administered simultaneously with seeding. After a 7-day incubation, the number of wells without spheres at each seeding density was determined and plotted against the number of cells per well.

### Statistical analysis

All experiments were repeated at least three times, and the data are presented as means ± s.e.m. The differences between two groups were assessed using unpaired two-tailed Student’s *t*-tests. For more than two groups, differences were determined using one-way analysis of variance (ANOVA) followed by a Bonferroni multiple comparison test. The relationships between variables were determined using Pearson’s correlation coefficient, after confirming that the data met the assumptions of normality and homogeneity of variances. Statistical significance was set at *P* < 0.05 (*P < 0.05, **P < 0.01, ***P < 0.001 and ****P < 0.0001). All statistical analyses were performed using GraphPad Prism v.7.0.

## Results

### DHA inhibits CSC characteristics and malignant behavior in OC

**Fig. 1:**
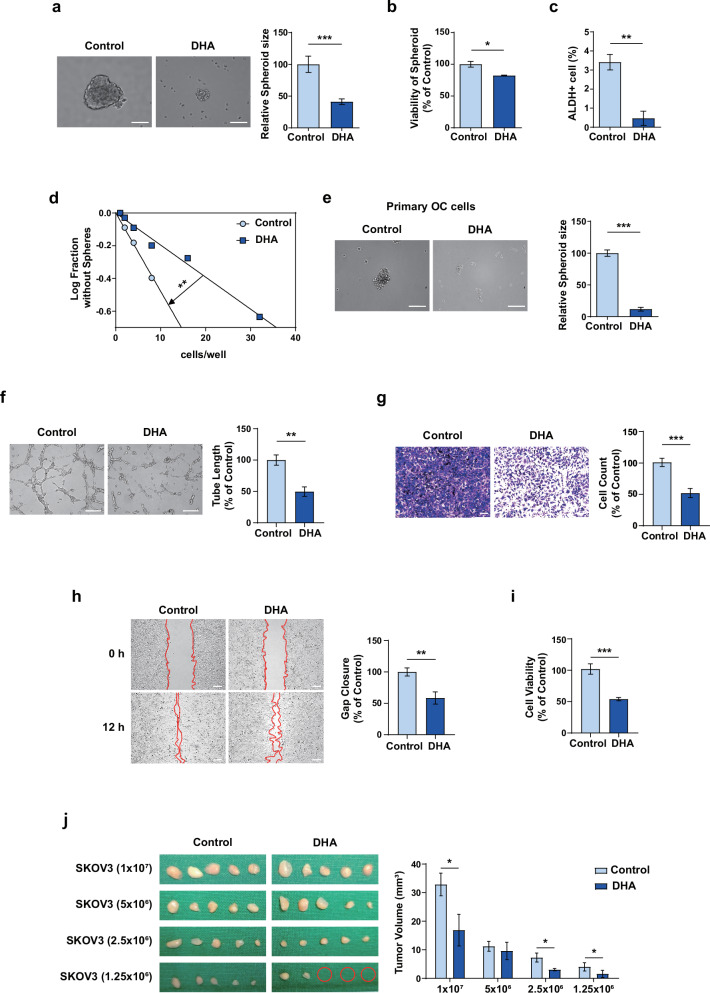
DHA inhibits CSC characteristics, VM, angiogenic properties and tumor growth. **a** The representative images of SKOV3 spheroids. The spheroids formed from SKOV3 cells treated with 50 µM DHA were significantly smaller than control spheroids. Scale bar, 100 μm. **b** The viability of SKOV3 spheroids after treatment with 50 μM DHA. **c** Th fluorescence-activated cell sorting of ALDH1-positive SKOV3 cells after treatment with 50 μM DHA. *P < 0.05, **P < 0.01 and ***P < 0.001 compared with controls assessed using unpaired two-tailed Student’s *t-*test in **a**–**c**. The error bars represent the s.e.m. **d** The limiting dilution assay showing the decreased self-renewal activity of SKOV3 spheroids after treatment with 50 μM DHA. **P < 0.01 for groups (control, DHA), cells per well and groups × cells per well by two-way ANOVA with Bonferroni’s multiple comparison test. **e** The representative images of spheroids formed from primary OC cells. The spheroids formed from primary OC cells treated with 50 µM DHA were significantly smaller than the control spheroids. Scale bar, 100 μm. **f** Inhibition of VM formation following the DHA treatment of SKOV3 cells in Matrigel. Scale bar, 500 μm. **g** The representative images of the invasion assay after DHA treatment of SKOV3 cells. Scale bar, 100 μm. **h** The representative images of the migration assay after DHA treatment of SKOV3 cells. Scale bar, 100 μm. **i** The viability of adherent SKOV3 cells after treatment with DHA. **j** The incidence and size of subcutaneous tumors in nude mice 14 days after injection with control or DHA-treated cells. The graph shows significantly decreased tumor size in mice injected with DHA-treated cells compared with mice injected with control cells. *P < 0.05, **P < 0.01 and ***P < 0.001 compared with controls by unpaired two-tailed Student’s *t*-test in **e**–**j**. The error bars represent the s.e.m.

To investigate whether DHA is involved in the regulation of CSC-like characteristics, we initially tested the effect of DHA on the spheroid-formation ability of SKOV3 cells and found that DHA treatment markedly suppressed spheroid formation (Fig. 1a). By contrast, CBP did not exhibit a significant effect on spheroid formation, suggesting that DHA is a promising candidate for overcoming the therapeutic limitations of CBP-based chemotherapy (Supplementary Fig. 1). Furthermore, DHA treatment significantly decreased the viability of the spheroid cells (Fig. 1b). Considering that ALDH1 activity is a hallmark of ovarian CSCs, we analyzed ALDH1 activity in SKOV3 cells. Treatment with DHA significantly decreased the percentage of ALDH1-positive SKOV3 spheroids (Fig. 1c). Moreover, the self-renewal potential, another typical trait of CSCs, was determined using an in vitro limiting dilution assay. DHA treatment significantly induced the serial exclusion of self-renewing cells (Fig. 1d), suggesting that the self-renewal activity of ovarian CSCs was inhibited. To further identify the effect of DHA on spheroid formation, we isolated fresh primary OC cells from human ovarian tumors and confirmed the suppressive effect of DHA on their spheroid formation (Fig. 1e). Considering that VM is significantly associated with CSCs and malignant tumors^30,31^, we examined VM formation by SKOV3 cells following DHA treatment. As shown in Fig. 1f, DHA treatment significantly inhibited VM formation by SKOV3 cells, suggesting that DHA can inhibit the aggressiveness of OC cells. Subsequently, we investigated the effects of DHA on the malignant attributes of SKOV3 cells. DHA treatment significantly reduced the migration, invasion and proliferation of SKOV3 cells (Fig. 1g–i). Finally, we evaluated the effect of DHA on tumor growth in vivo. SKOV3 xenografts in nude mice treated with DHA resulted in fewer tumors and a markedly smaller tumor size than those in control mice (Fig. 1j), further validating our in vitro findings that DHA exerts antitumor effects. Collectively, these findings suggest that DHA attenuates CSC-like characteristics and malignant behavior in OC cells and thus may be a potential therapeutic agent for overcoming the limitations (for example, chemoresistance) of current therapies in treating OC.

### DHA inhibits CSC-like traits by attenuating BMI-1 in OC cells

**Fig. 2:**
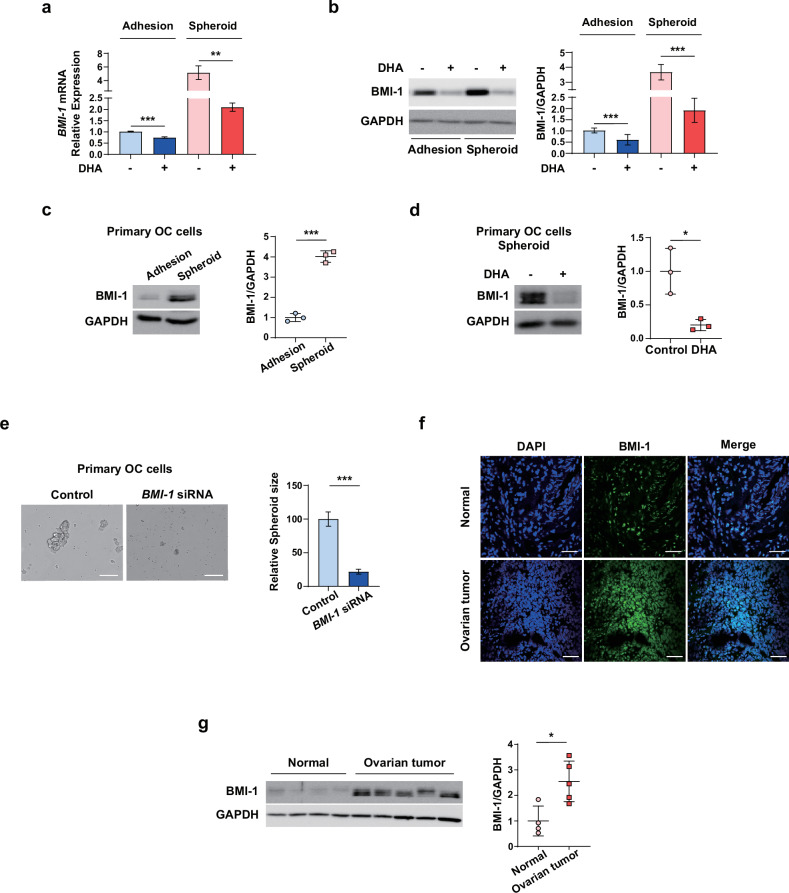
DHA inhibits ovarian CSC properties by modulating BMI-1 expression. **a**, **b** The mRNA (**a**) and protein (**b**) expression of BMI-1 in the adherent and spheroid SKOV3 cells after treatment with 50 μM DHA. **c** The BMI-1 protein expression in spheroids formed from primary OC cells. **d** The BMI-1 protein expression in spheroids formed from primary OC cells after treatment with 50 µM DHA. **e** The representative images of spheroids formed from primary OC cells transfected with *BMI-1* siRNA. The spheroids transfected with *BMI-1* siRNA were significantly smaller than those transfected with control siRNA. Scale bar, 100 μm. **f**, **g** The histopathological (**f**) and western blot analyses (**g**) revealed upregulated BMI-1 expression in ovarian tumor tissues compared with that in normal tissues. Scale bar, 50 μm. *P < 0.05, **P < 0.01 and ***P < 0.001 for comparisons tested using unpaired two-tailed Student’s *t*-tests. The error bars indicate the s.e.m.

To identify the mechanism underlying the anti-CSC effect of DHA in OC, we used a human CSC marker quantitative polymerase chain reaction array. The SKOV3 spheroids were exposed to CBP or DHA, and the expression of CSC markers was analyzed. The CBP treatment increased the expression of CSC markers, including *BMI-1* (Supplementary Fig. 2), suggesting the involvement of BMI-1 in CSC-associated CBP resistance in OC. Meanwhile, the DHA treatment markedly reduced the expression of most CSC markers, especially *BMI-1*, in the CSC-enriched spheroids (Supplementary Fig. 2). Next, we examined BMI‑1 expression in adherent SKOV3 and OVCAR3 cells treated with CBP or DHA before evaluating its expression in spheroids. The CBP treatment did not alter BMI‑1 expression, whereas DHA treatment caused a dose-dependent reduction (Supplementary Fig. 3a, b). We then compared BMI‑1 expression between spheroids and adherent cells. The quantitative polymerase chain reaction and western blot experiments confirmed that BMI-1 expression was markedly increased in SKOV3 spheroids compared with their adherent counterparts and was downregulated by DHA treatment in spheroid SKOV3 cells (Fig. 2a, b). Similarly, the expression of BMI-1 was markedly increased in spheroids derived from primary OC cells freshly isolated from human ovarian tumors and subsequently decreased following DHA treatment (Fig. 2c, d). By contrast, CBP treatment did not significantly influence BMI-1 expression in SKOV3 spheroids (Supplementary Fig. 3c).

Considering these findings, we investigated the effects of BMI-1 knockdown on SKOV3 spheroids. Consistent with the results of DHA treatment, *BMI-1* knockdown markedly decreased spheroid formation (Fig. 2e). Furthermore, histopathological and western blot analyses revealed that BMI-1 expression was higher in ovarian tumor tissues than in normal tissues (Fig. 2f, g). These findings suggest that BMI-1 plays a key role in CBP-mediated chemoresistance and that DHA, which inhibits the expression of CSC markers, including BMI-1, may be a potential drug to overcome the limitations of current therapies by broadly suppressing CSC traits in OC.

### DHA inhibits VEGF-A-mediated tumor neovascularization in OC cells

**Fig. 3:**
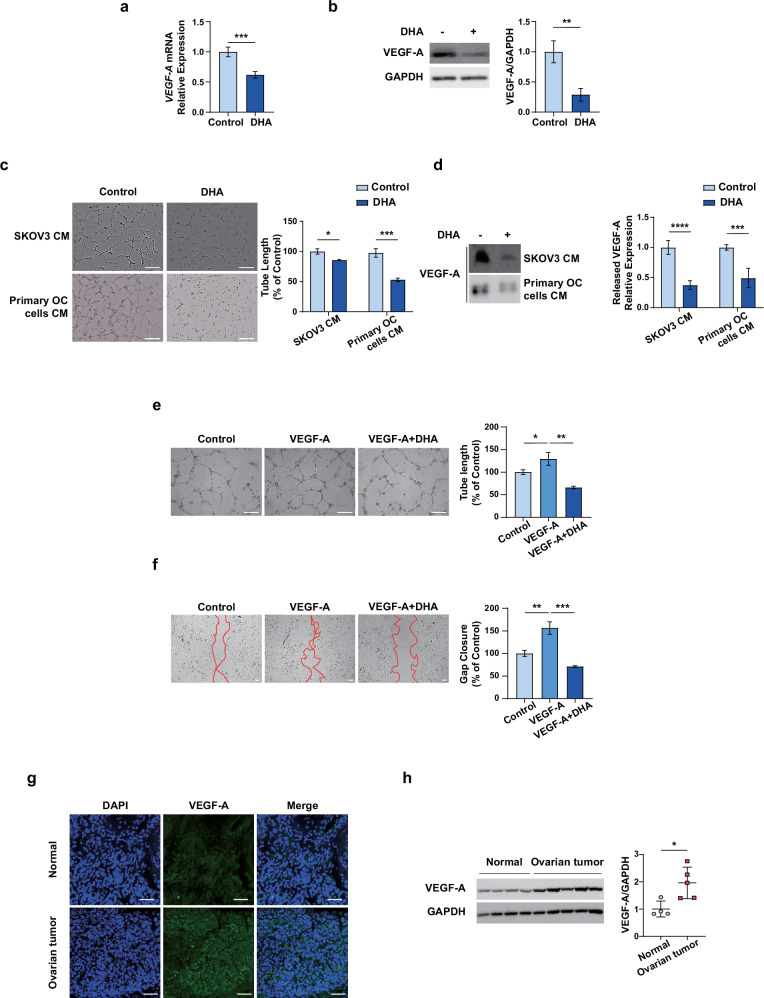
DHA attenuates angiogenic properties by inhibiting VEGF in OC cells. **a**, **b** VEGF-A mRNA (**a**) and protein (**b**) expression in adherent SKOV3 cells after treatment with 50 μM DHA. **c** The endothelial tube formation in CM, demonstrating cancer-mediated paracrine function with or without DHA treatment. The HUVECs were plated onto a Matrigel layer and treated with CM from SKOV3 cells or primary OC cells treated with DHA. Scale bar, 500 μm. **d** The VEGF-A in the CM was concentrated using Amicon ultracentrifugal filters before performing a western blot. **e** The representative images of the tube-formation assay after the treatment with VEGF-A and 50 μM DHA in ECs. Scale bar, 500 μm. **f** The representative images of the migration assay after the treatment with VEGF-A and 50 μM DHA in ECs. Scale bar, 200 μm. **g**, **h** The histopathological (**g**) and western blot analyses (**h**) revealed that VEGF-A expression was upregulated in ovarian tumor tissues compared with normal tissues. *P < 0.05, **P < 0.01, ***P < 0.001 and ****P < 0.0001 compared with controls by unpaired two-tailed Student’s *t-*test or one-way ANOVA with Bonferroni’s multiple comparison test. The error bars represent the s.e.m.

VEGF-A is frequently overexpressed in OC and is involved in ovarian tumor neovascularization^40^. Thus, we explored whether DHA can suppress VEGF-A expression and consequently inhibit VEGF-A-mediated angiogenesis. Similar to its regulatory effect on BMI-1 expression, the DHA treatment reduced the mRNA and protein expression of VEGF-A in SKOV3 and OVCAR3 cells (Fig. 3a, b and Supplementary Fig. 4a, b), whereas the CBP treatment had no effect on VEGF-A expression in SKOV3 cells (Supplementary Fig. 4c). Next, we evaluated whether the DHA treatment of OC cells affects the angiogenic properties of endothelial cells (ECs) in a paracrine manner. ECs were incubated in Matrigel-containing cell-free supernatant obtained from OC cells treated with DHA or CBP. As shown in Fig. 3c, the conditioned medium (CM) collected from the DHA-treated SKOV3 and primary OC cells inhibited EC tube formation in Matrigel, indicating a suppression of angiogenesis. By contrast, the CM from the CBP-treated cells did not significantly influence EC tube formation (Supplementary Fig. 4d). The proteins in the CM were concentrated using Amicon ultracentrifugal filters to examine the effect of DHA on VEGF-A levels in the CM. The western blot analysis revealed that the amount of VEGF-A released into the CM of the DHA-treated cells was lower than that released into the CM of the DMSO-treated control cells (Fig. 3d). These data indicate that the antiangiogenic role of DHA is mediated, at least in part, by the inhibition of VEGF-A expression and secretion in OC. To further determine whether DHA regulates the VEGF-A-mediated angiogenic properties of ECs, we performed in vitro tube formation and migration assays with ECs. The DHA treatment significantly reduced the tube formation and migration of ECs and significantly prevented the VEGF-A-induced tube formation and migration of ECs (Fig. 3e, f). Finally, histopathological and western blot analyses revealed that VEGF-A expression was higher in ovarian tumor tissues than in normal tissues (Fig. 3g, h). Collectively, these results suggest that DHA attenuates ovarian tumor neovascularization by targeting VEGF-A.

### DHA-mediated miR-200b modulates CSC characteristics by regulating BMI-1 expression in OC cells

**Fig. 4:**
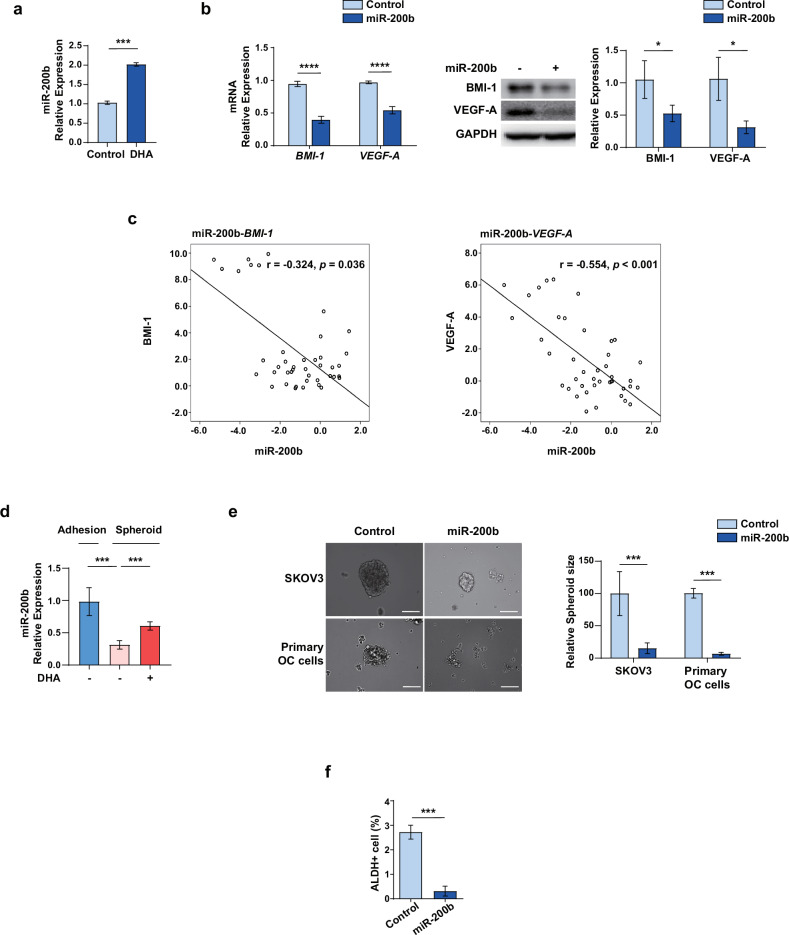
DHA-mediated miR-200b modulates ovarian CSC properties by altering BMI-1 expression. **a** The miR-200b expression in OC cells treated with 50 μM DHA. **b** The BMI-1 and VEGF-A mRNA and protein expression in OC cells transfected with miR-200b. **c** The inverse correlation between *BMI-1* and *VEGF-A* mRNA expression and miR-200b expression in ovarian tumor tissues. The relationships between variables were determined by the Spearman’s correlation coefficient. **d** The miR-200b expression in adherent and spheroid OC cells after treatment with 50 µM DHA. **e** The representative images of primary OC cells and SKOV3 spheroids transfected with miR-200b. The spheroids formed from primary OC cells and SKOV3 cells transfected with miR-200b were significantly smaller than those formed from control cells. Scale bar, 100 μm. **f** The fluorescence-activated cell sorting of ALDH1-positive OC cells transfected with miR-200b. *P < 0.05, ***P < 0.001 and ****P < 0.0001 compared with controls by unpaired two-tailed Student’s *t*-test or one-way ANOVA with Bonferroni’s multiple comparison test. The error bars represent the s.e.m.

Following the above results, we further investigated the mechanisms underlying the effects of DHA treatment. Micro RNAs (miRNAs) play key roles in diverse cellular processes, including CSC phenotypes, tumor progression, drug resistance and angiogenesis, owing to their capacity to target multiple mRNAs^41,42^. We hypothesized that the dual regulation of BMI-1 and VEGF-A expression by DHA is mediated by miRNAs (that is, miR-15, miR-200b and miR-424) that reportedly affect the stability of BMI-1 and VEGF-A mRNAs^43,44^. Among these, miR-200b exhibited a significantly increased expression in OC cells after DHA treatment (Fig. 4a). Therefore, we examined the effects of miR-200b overexpression on BMI-1 and VEGF-A expression in OC cells. Consistent with the findings of previous studies, miR-200b overexpression significantly decreased the mRNA and protein levels of BMI-1 and VEGF-A in OC cells (Fig. 4b). To further investigate the interaction between miR-200b and its target genes, we performed a comprehensive analysis of miR‑200b and *BMI-1* and *VEGF-A* levels in clinical ovarian tumor tissues that were collected and analyzed using matched mRNA and miRNA expression profiles, revealing a significant inverse correlation between miR-200b expression and both *BMI-1* and *VEGF-A* expression (Fig. 4c). miR-200b is a tumor suppressor in several types of cancer, and low miR-200b expression is notably associated with CSCs, chemoresistance and poor prognosis^45–47^. Accordingly, we examined whether miR-200b expression was affected in OC spheroids. The expression level of miR-200b in OC spheroids was significantly decreased but was restored following DHA treatment in a dose-dependent manner (Fig. 4d), suggesting a important involvement of miR-200b in the regulation of DHA-mediated BMI-1 expression. To investigate the functional role of the miR-200b-BMI-1 axis regulated by DHA, we examined whether miR-200b regulates the CSC properties of OC cells. miR-200b overexpression significantly inhibited the spheroid-formation ability of SKOV3 and primary OC cells (Fig. 4e). In addition, the percentage of ALDH1-positive cells was lower in miR-200b-overexpressing spheroids than in controls (Fig. 4f). These results indicate that DHA-mediated miR-200b expression regulates CSC characteristics, at least in part, by inhibiting BMI-1 expression in OC.

### DHA-mediated miR-200b modulates tumor neovascularization by impeding VEGF-A/VEGFR2-mediated microenvironmental crosstalk between OC cells and ECs

**Fig. 5:**
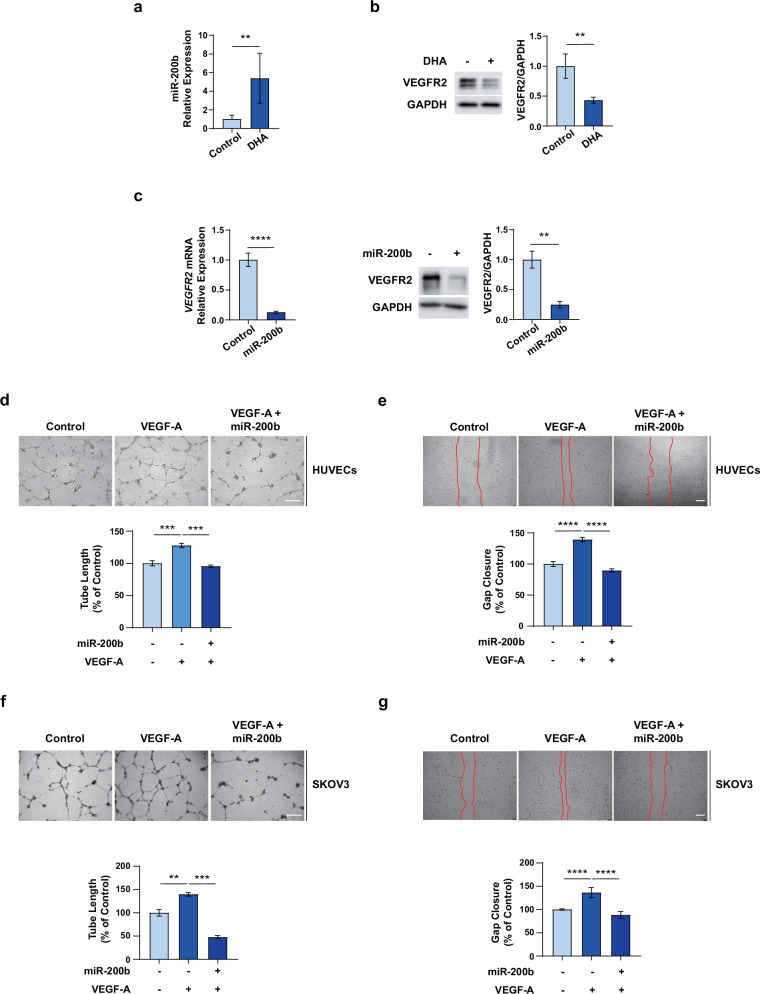
DHA-mediated miR-200b regulates VEGF-A-mediated tumor neovascularization by modulating VEGFR2 expression in ECs. **a** The miR-200b expression in ECs in response to treatment with 50 μM DHA. **b** The VEGFR2 protein expression in ECs in response to treatment with 50 µM DHA. **c** The VEGFR2 mRNA and protein expression in ECs transfected with miR-200b. **d** The representative images of the tube-formation assay after treatment of ECs with VEGF-A and transfection with miR-200b. Scale bar, 500 μm. **e** The representative images of the migration assay after treatment of ECs with VEGF-A and transfection with miR-200b. Scale bar, 200 μm. **f** The representative images of the tube-formation assay after treatment with VEGF-A and miR-200b transfection in SKOV3 cells. Scale bar, 500 μm. **g** The representative images of the migration assay after treatment with VEGF-A and miR-200b transfection in SKOV3 cells. Scale bar, 200 μm. **P < 0.01, ***P < 0.001 and ****P < 0.0001 compared with controls by unpaired two-tailed Student’s *t*-test or one-way ANOVA with Bonferroni’s multiple comparison test. The error bars represent the s.e.m.

VEGF-A promotes angiogenic properties mostly by binding to VEGFR2. Therefore, we examined whether DHA regulates miR-200b and VEGFR2 in ECs in the context of OC. The DHA treatment markedly increased miR-200b expression (Fig. 5a) and decreased VEGFR2 expression (Fig. 5b) in ECs. Furthermore, miR-200b overexpression significantly decreased the mRNA and protein expression levels of VEGFR2 in ECs (Fig. 5c), suggesting that DHA reversed VEGF-A-mediated paracrine effects by regulating the miR-200b–VEGFR2 axis in ECs. To investigate whether miR-200b regulates ovarian tumor neovascularization, we examined the effect of miR-200b on the VEGF-A-mediated angiogenic properties of ECs. miR-200b significantly inhibited the VEGF-A-induced tube formation and migration of ECs (Fig. 5d, e). Furthermore, miR-200b overexpression significantly decreased the VEGF-A-mediated VM formation and migration of SKOV3 cells (Fig. 5f, g). Collectively, these results indicate that DHA-mediated miR-200b inhibits tumor neovascularization, at least in part, by inhibiting VEGFR2 expression in ECs.

### Combined treatment with DHA and CBP impedes OC growth and peritoneal seeding in a mouse model of intraperitoneal OC

**Fig. 6:**
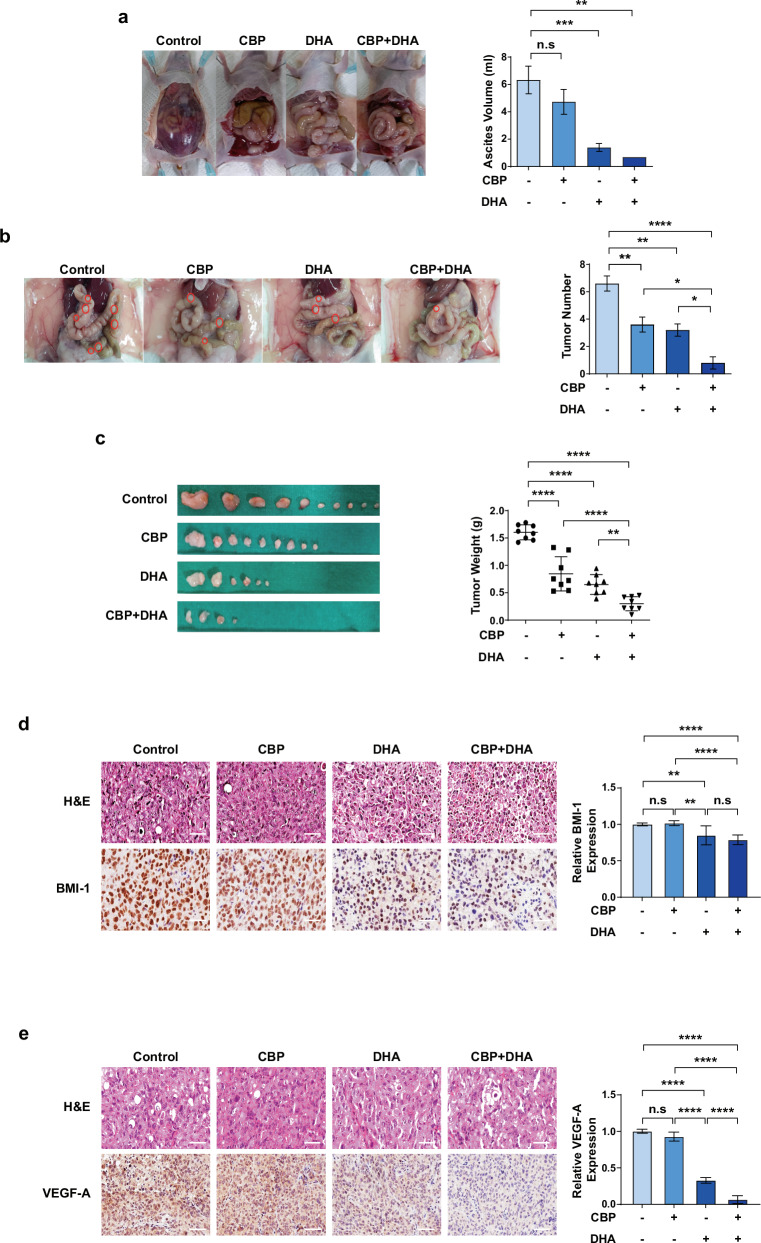
Combined treatment with DHA/CBP inhibits OC in an intraperitoneal OC murine model. **a** The representative images depicting the extent of ascites formation in OC-bearing mice treated with control (vehicle), CBP, DHA or a combination of CBP and DHA. **b** The representative images of the peritoneal cavities of mice showing a reduction in peritoneal tumors in the CBP, DHA and CBP + DHA treatment groups compared with those in the control group. The quantification of peritoneal metastases is shown. **c** The dissected ovarian tumors from each experimental group, with the measured tumor weights represented in the adjacent graph. **d**, **e** The histological analysis of ovarian tumors using hematoxylin and eosin staining, and the BMI-1 (**d**) and VEGF-A (**e**) expression levels determined using IHC staining. Right: the details of the quantitative expression are shown on the graph. Scale bars, 50 mm. *P < 0.05, **P < 0.01, ***P < 0.001 and ****P < 0.0001 compared with controls by unpaired two-tailed Student’s *t*-test or one-way ANOVA with Bonferroni’s multiple comparison test. The error bars represent the s.e.m.

Our findings showed that DHA may be a potential therapeutic agent to overcome the limitations of CBP by reducing BMI-1, VEGF and VEGFR2 levels, which are associated with CSCs and tumor neovascularization and are crucial for chemoresistance. Therefore, we investigated the combined effects of DHA and CBP treatment on OC growth and peritoneal seeding in an intraperitoneal OC model. First, the body weight of each mouse was monitored throughout the experimental period to determine the safety of DHA, CBP and their combination. The different treatment groups showed no significant differences in body weight, indicating that the combination of DHA and CBP had no obvious side effects (Supplementary Fig. 5a). An analysis of blood samples revealed no significant differences in the white blood cell count, an essential component of the immune system (normal range: 2000–10,000 µl), in the treatment groups compared with the control group. In addition, no significant variations were noted in the levels of hemoglobin (normal range: 14–15 g/dl), confirming that neither the individual nor combination treatments influenced the oxygen-carrying capacity of the blood. Moreover, the platelet count (0.8 × 10^6^–1.6 × 10^6^ per microliter) remained consistent following both individual and combined DHA treatments (Supplementary Fig. 5b). In addition, no notable differences were found in the levels of AST (reflecting liver function), ALP (indicating liver metabolism) and ALT compared with the control group. Finally, an assessment of kidney toxicity by examining creatinine levels revealed no significant differences between the DHA-treated and control groups (Supplementary Fig. 5c). In summary, these findings suggest that DHA has minimal effects on physiological indicators in mice that received individual or combined treatments, confirming its high safety profile. Considering that ascites contributes to intraperitoneal tumor dissemination, we investigated the formation of ascites in the intraperitoneally inoculated mice. Ascites formation was significantly inhibited in the CBP and DHA treatment groups compared with the control group, and their combined treatment substantially abrogated ascites formation (Fig. 6a). In addition, peritoneal seeding in the intraperitoneal tumor model was substantially reduced in the DHA and CBP combination group compared with the control group (Fig. 6b). Both DHA and CBP inhibited tumor growth even when administered alone; however, their combination resulted in an even greater suppression of tumor growth (Fig. 6c). Finally, we showed that BMI-1 and VEGF-A expression in the tumors was decreased in the DHA and DHA + CBP combination groups, but was not affected in the CBP group (Fig. 6d, e). Together, these results suggest that DHA can improve CBP sensitivity through the inhibition of typical CSC properties by regulating BMI-1 and VEGF-A expression, thereby preventing the peritoneal metastasis of OC. Further studies are required to determine the efficacy and optimal dose of DHA for clinical applications.

### High expression of BMI-1 and VEGF-A is associated with poor prognosis in human OCs

**Fig. 7:**
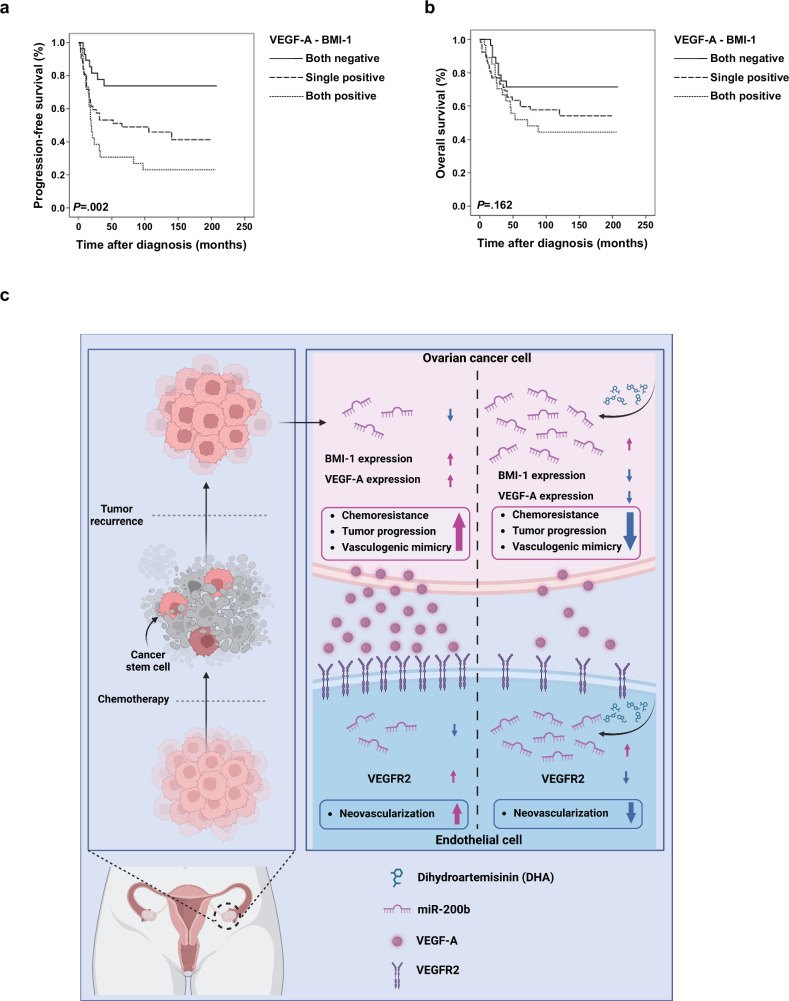
Kaplan–Meier estimates of PFS and OS according to VEGF-A and BMI-1 status. **a**, **b** The PFS (**a**) and OS (**b**) with their corresponding *P* values were calculated using log-rank analysis. **c** A schematic summary of the proposed mechanism of action of DHA in suppressing OC and inhibiting neovascularization, including molecular targets and affected pathways. This diagram encapsulates the interactions between the treatment and the pathophysiological processes of OC.

We investigated the prognostic value of VEGF-A and BMI-1 in 107 patients diagnosed with OC (Supplementary Table 1). Most cases (two thirds) were histologically classified as high grade (76.6%) and serous adenocarcinoma (57.0%). According to the International Federation of Gynecology and Obstetrics (FIGO) stage, OCs were mainly diagnosed at advanced clinical stage III–IV (62.6%). Most patients with OC, except for the subgroup of patients with early stage disease, were treated with platinum-based adjuvant chemotherapy (84.1%). The IHC analysis showed that of the 107 patients, 76 (71.0%) were positive for VEGF-A staining, and 29 (27.1%) were positive for BMI-1 staining. The relationships between clinicopathological factors and VEGF-A and BMI-1 expression levels in patients with OC are presented in Supplementary Table 1. In the present study, VEGF-A and BMI-1 expression showed no significant correlation with age, FIGO stage, histological type, histological grade, cancer antigen 125 (CA-125) levels, postoperative residual mass or adjuvant chemotherapy. Next, we investigated the prognostic value of VEGF-A and BMI-1 expression in predicting PFS in OC using a multivariate Cox regression analysis adjusted for age, FIGO stage, histological type, histological grade, CA-125 levels and residual mass (Table 1). VEGF-A and BMI-1 expression are independent risk factors for PFS. VEGF-A positivity significantly increased the risk by 2.208 times compared with VEGF-A negativity (confidence interval (CI): 1.001–4.872, *P* value: 0.043), whereas BMI-1 positivity significantly increased the risk by 2.167 times compared with BMI-1 negativity (CI: 1.178–3.986, *P* value: 0.013). To determine whether VEGF-A and BMI-1 co-expression could affect the prognosis of patients with OC, we divided the patients into three groups: group A, patients positive for both variables (VEGF-A^+^ and BMI-1^+^); group B, patients positive for at least one of the variables (VEGF-A^+^ and BMI-1^−^ or VEGF-A^−^ and BMI-1^+^); and group C, patients negative for both variables (VEGF-A^−^ and BMI-1^−^). The Kaplan–Meier analysis revealed a significant difference in PFS probability among the groups (*P* value of log-rank test: 0.002) (Fig. 7a), but no significant difference in OS probability among them (*P* value of log-rank test: 0.162) (Fig. 7b). Furthermore, a detailed analysis of the risk of PFS using a multiple Cox model showed that, compared with the hazard ratio of VEGF-A and BMI-1 double negativity, that of VEGF-A single positivity (VEGF-A^+^, BMI-1^−^) was 2.158 times higher (CI: 0.929–5.009; *P* value: 0.043), that of BMI-1 single positivity (VEGF-A^−^, BMI-1^+^) was 3.602 times higher (CI: 1.460–7.096; *P* value: 0.036) and that of double positivity (VEGF-A^+^, BMI-1^+^) was 3.863 times higher (CI: 1.602–9.312; *P* value: 0.003) (Supplementary Table 2). These results confirm that VEGF-A+ and BMI-1+ are independent prognostic factors for PFS and that VEGF-A^+^ and BMI-1^+^ double-positive patients have the worst prognosis. However, these results were not validated for OS. Moreover, an analysis of The Cancer Genome Atlas dataset further demonstrated that low miR‑200b expression was significantly associated with shorter OS (*P* = 0.033), whereas high BMI‑1 expression was strongly correlated with poor OS (*P* = 0.00035) (Supplementary Fig. 6a, b). Together, these clinical and The Cancer Genome Atlas-based analyses highlight the prognostic significance of the VEGF‑A–BMI‑1 axis and further underscore the contribution of the miR‑200b–BMI‑1 pathway to OC progression.

**Table 1:** Univariate and multivariate analyses of variables associated with PFS in patients with OC.

Variable	Univariate		Multivariate	
	HR (95% CI)	*P*	HR (95% CI)	*P*
Age (years) (>51^a^ versus ≤51)	1.615 (0.937–2.784)	0.084	–	–
FIGO stage (III–IV versus I–II)	5.211 (2.528–10.741)	<0.001	4.235 (1.945–9.220)	<0.001
Histology type (serous versus nonserous^b^)	1.764 (0.990–3.141)	0.054	–	–
Histologic grade (2–3 versus 1)	2.092 (1.021–4.284)	0.044	–	–
CA-125 (unit per milliliter) (>646^c^ versus ≤646)	1.887 (1.051–3.388)	0.034	–	–
Residual mass (>1cm versus ≤1 cm)	2.831 (1.503–5.333)	0.006	2.105 (1.085–4.086)	0.028
VEGF-A (positive versus negative)	3.140 (1.480–6.663)	0.003	2.208 (1.001–4.872)	0.043
BMI-1 (positive versus negative)	2.106 (1.213–3.659)	0.008	2.167 (1.178–3.986)	0.013

^a^Mean age; ^b^mucinous, endometroid and clear cell histology; ^c^mean level. *HR* hazard ratio.

## Discussion

OC is the most fatal type of gynecological cancer and is usually diagnosed after the disease has progressed to an advanced stage^48^. Interestingly, OC has a different mechanism of metastasis compared with other cancers; exfoliated ovarian tumor cells are transported throughout the peritoneum by the peritoneal fluid and are disseminated within the abdominal cavity^49^. Intraperitoneal implants in patients with advanced-stage disease are the result of single cells and multicellular spheroids that adhere to the mesothelial lining of various abdominal organs to establish secondary lesions^50^. Although most patients are highly responsive to standard treatments involving surgical tumor debulking and platinum-based chemotherapy, long-term survival and cure rates are rather low owing to the acquisition of resistance over time, leading to tumor recurrence. This phenomenon is commonly attributed to a subpopulation of cancer cells, namely, ovarian CSCs, which are closely associated with the chemoresistance, recurrence and metastasis of OC^51^. Furthermore, CSCs are a vital source of tumor neovascularization inducers, including VEGF, which drives angiogenesis and VM and contributes to tumor progression and metastasis^52^. Accumulating data have provided unequivocal evidence that anti-CSC and antitumor neovascularization therapies have therapeutic benefits for malignant tumors^53,54^. Therefore, it is imperative to identify novel and improved drugs that are able to reduce tumor recurrence and metastasis in OC by targeting CSCs. The use of DHA, an antimalarial drug, has now been extended to various disease models, such as inflammatory disease^55^, Alzheimer’s disease^56^, steatosis^57,58^ and organ fibrosis^59,60^. In particular, DHA has been considered a potential anticancer agent for many types of cancer because of its antiproliferative and antiangiogenic activities, as well as its extremely low toxicity^61^. However, the mechanisms underlying the activity of DHA in OC progression and its effect on overcoming drug resistance, OC relapse and metastasis remain largely unknown. In the current study, we report that DHA treatment efficiently inhibited the action of CSCs, angiogenesis, VM and tumor resistance to CBP via an miRNA-dependent mechanism in OC. The major findings of this study are as follows: (1) DHA, but not CBP, inhibits CSC characteristics and malignant behavior by targeting BMI-1 and VEGF-A in OC; (2) DHA regulates miR-200b expression, which in turn targets key factors of CSC and tumor neovascularization regulation, namely, BMI-1 and VEGF-A/VEGFR2, respectively; (3) an analysis of clinical data from patients with OC revealed that VEGF-A and BMI-1 double positivity are independent prognostic factors for PFS and that VEGF-A and BMI-1 double positivity indicate the worst prognosis; and (4) the combination of DHA and CBP impedes OC growth and peritoneal seeding in a mouse model of intraperitoneal OC. These findings may expand the clinical applications of DHA to not only suppress tumor growth but also inhibit CSCs, thereby overcoming the therapeutic limitations of current chemotherapies (Fig. 7c).

BMI-1 plays an important role in the chemoresistance of OC cells by regulating reactive oxygen species and glutathione levels, thus conferring protection against chemotherapeutic molecules^62^. Moreover, its expression is upregulated through multiple mechanisms^20,62,63^. In CSCs, the high expression of BMI-1^19^ is associated with spheroid formation. We found that BMI-1 was highly expressed in the OC spheroids, which were enriched for CSC populations. Surprisingly, CBP treatment had no effect on BMI-1 expression in ovarian spheroids, whereas DHA treatment markedly reduced the expression of BMI-1 and several other CSC markers, which reduced the number of spheroids, attenuated CSC self-renewal activity and cancer growth and improved CBP sensitivity. The combined treatment with DHA and CBP substantially suppressed tumor growth and peritoneal seeding in the intraperitoneal ovarian tumor model. Furthermore, our clinical data from patients with OC showed that high levels of BMI-1 were associated with poor PFS. These results suggest that BMI-1 is implicated in CSC-associated drug resistance and that DHA-mediated BMI-1 inhibition is effective against metastatic OC and can overcome cancer chemoresistance to platinum-based drugs.

Accumulating evidence suggests that vascular niches are essential for maintaining the CSC traits that are crucial for tumor neovascularization^64^. The stemness-related transcription factor KLF4 induces CSC characteristics in osteosarcoma^65^ and promotes angiogenesis in ECs by regulating the Notch signaling pathway. VEGF-A is markedly overexpressed in OC^35^, and CSCs directly participate in tumor neovascularization by producing high levels of VEGF^66^. Furthermore, BMI-1 has been implicated in glioma angiogenesis by upregulating VEGF expression^67^, and VEGF–NRP2 signaling is required to maintain BMI-1 expression^68^.

In contrast to previous studies, our clinical data from patients with OC demonstrated that neither BMI-1 nor VEGF-A positivity was related to an advanced stage or tumor grade. However, in multivariate Cox regression analysis, positivity for each gene was found to be a poor prognostic factor for PFS. Interestingly, the hazard ratio for PFS was worst in the case of double positivity. In addition, the statistical significance between BMI-1 and VEGF-A positivity was confirmed using logistic regression analysis. These results support the relationship between BMI-1 and VEGF-A in OC progression.

Tumors require vascularization to grow and metastasize; angiogenesis, as well as alternative mechanisms, such as VM, provide the necessary oxygen and nutrients. CSC markers such as CD133 and ALDH1 have been closely associated with VM, advanced tumor stage, chemoresistance and poor prognosis in OC^69^. VEGF-A promotes VM formation in OC, highlighting the importance of VM-targeting strategies through the regulation of VEGF-A^70^. In this study, DHA treatment significantly inhibited the angiogenic properties of ECs by suppressing VEGF secretion and VEGFR2 expression in OC cells and ECs, respectively. Moreover, DHA treatment significantly decreased VEGF-A-mediated VM formation by OC cells. Thus, DHA inhibits angiogenesis and VM, suggesting that it exerts its therapeutic effects by targeting VEGF signaling. However, further studies are warranted to clarify the underlying molecular mechanisms of the association between BMI-1 and VEGF-A in ovarian CSCs and angiogenesis.

miRNAs are involved in the maintenance of CSC properties, drug resistance and tumor neovascularization^71–73^. In our previous study, we showed that miR-424 and miR-503 inhibit CSC properties by directly targeting WEE1 in OC, thereby overcoming chemoresistance and suppressing peritoneal implantation metastasis. In addition, miR-15, miR-16, miR-200b and miR-424 target BMI-1, VEGF-A and VEGFR2^74^. In the present study, we screened these miRNAs to determine whether they were regulated by DHA and found that DHA treatment significantly increased miR-200b expression in OC spheroids and ECs. This is consistent with previous studies, which showed that miR-200b expression is downregulated in most OCs and that low miR-200b expression is closely associated with chemoresistance and poor prognosis in various types of cancer^47,75^. However, the specific roles of miR-200b in CSCs and neovascularization in OC remain unclear. Our current findings demonstrate that DHA-mediated miR-200b expression plays an essential role in regulating CSC characteristics and malignant behavior in OC, partly by inhibiting VEGF-A and BMI-1 expression. VEGFR2, the receptor for the VEGF-A ligand secreted by OC cells, is highly expressed in ECs, facilitating the autocrine and paracrine effects of VEGF-A in the ovarian tumor microenvironment^76^. However, further studies are warranted to elucidate the mechanisms and functional roles of DHA-mediated upregulation of miR-200b expression in ECs.

This study showed that DHA treatment could reverse VEGF-A-mediated paracrine effects by regulating the miR-200b–VEGFR2 axis in ECs. Moreover, we found that DHA-mediated miR-200b can inhibit the VEGF-A-mediated angiogenic properties of ECs. These findings suggest a novel role for DHA in OC–EC crosstalk, which may be crucial in OC progression. Collectively, the results showed that DHA-mediated miR-200b inhibits CSC characteristics, angiogenesis and VM by directly downregulating VEGF-A, VEGFR2 and BMI-1 expression. However, further studies are required to elucidate the mechanisms by which DHA treatment induces miR-200b expression in OC cells and ECs. This study provides novel insights into the important roles of the miR-200b–BMI-1 and VEGF-A/VEGFR2 axis in regulating ovarian CSCs, tumor neovascularization and chemoresistance. DHA targets ovarian CSCs and tumor neovascularization, has low toxicity and is inexpensive, making it suitable for rapid clinical applications in OC treatment. We propose that DHA, in combination with current chemotherapeutic treatments, has potential applications in the treatment of OC relapses.

## Availability of data and materials

All materials are available from the corresponding author upon request.

## Supplementary information

Supplementary Information
