# Supplementary material for: Dihydroartemisinin inhibits metastatic potential and cancer stemness by modulating the miR-200b–BMI-1/VEGF-A axis in ovarian cancer
Source: Exp Mol Med. 2025 Dec 5;57(12):2782–97. doi: 10.1038/s12276-025-01582-2 (PMC12800336; doi:10.1038/s12276-025-01582-2)
Supplement: Supplementary file 1 — Supplementary Information [file 12276_2025_1582_MOESM1_ESM.pdf]

Supplementary Information

Dihydroartemisinin inhibits metastatic potential and cancer stemness by modulating the miR-200b–BMI-1/VEGF-A axis in ovarian cancer

This file includes:

- **Supplementary methods**
- **Supplementary figures 1 to 6**
- **Supplenentary tables 1 to 4**

Supplementary methods

Transfection

Lipofectamine RNAimax (Invitrogen, Carlsbad, CA, USA) was used for microRNA (miRNA) and small interfering RNA (siRNA) transfection following the manufacturer's instructions. Pre-miRNAs from Bioneer (Daejeon, South Korea) and siRNAs from Invitrogen were used for transfection. Validated human BMI-1 siRNAs (1299001) and the AccuTarget miRNA mimic has-miR-200b (SMM-001) were purchased from Invitrogen and Bioneer, respectively. Negative control siRNA was purchased from Invitrogen. AccuTarget miRNA mimic negative control #1 (Bioneer) and Stealth RNAi siRNA negative control (Invitrogen) were used as negative controls.

Western blotting

Cells were lysed using RIPA buffer (Biosesang, Yongin, South Korea) containing 1X protease inhibitor (Dawinbio, Hanam, South Korea). The lysed cells were centrifuged at 13,000 rpm and 4 °C for 15 min, and the protein content was quantified using a Pierce BCA Protein Assay kit (Thermo Fisher Scientific, Waltham, USA). Cell lysates were separated on sodium dodecyl sulfate-polyacrylamide gels and transferred onto polyvinyl difluoride membranes (Millipore, Burlington, MA, USA). The membranes were incubated with primary antibodies against BMI-1 (1:1000; Cell Signaling Technology, Danvers, MA, USA), glyceraldehyde 3-phosphate dehydrogenase (GAPDH; 1:4000; Cell Signaling Technology), VEGF-A (1:1000; Abcam, Cambridge, UK), and vascular endothelial growth factor receptor 2 (VEGFR2; Cell Signaling Technology) (Supplementary Table 3). Immunodetection was performed using horseradish peroxidase–conjugated secondary antibodies, and visualized using an enhanced chemiluminescence detection system (Amersham, Buckinghamshire, UK).

RNA extraction and quantitative reverse-transcriptase PCR (qRT-PCR)

Total RNA was isolated using the miRNeasy Kit for RNA isolation (Qiagen, Hilden, Germany). Purified mRNA was reverse transcribed using the UltraScript 2.0 cDNA Synthesis Kit (PCR Biosystems, London, UK). qRT-PCR was performed using the PCRBIO SyGreen Blue Mix Lo-ROX (PCR Biosystems) according to the manufacturer's instructions. The primer sequences used to assess mRNA expression are listed in Supplementary Table 4. For miRNA analysis, purified RNA was reverse transcribed using the TaqMan

MicroRNA Reverse Transcription Kit (Applied Biosystems, Foster City, CA, USA). qRT-PCR was performed using the TaqMan Universal Master Mix II (Applied Biosystems). MiR-200b was detected using TaqMan probes. The small RNA RNU6B was used as the internal control for data normalization.

Migration assay

SKOV3 cells and HUVECs were seeded in 12-well plates at a density of 2 × 10^5 cells/mL and cultured until confluence. The cells were treated with DHA or VEGF-A or transfected with miR-200b. Subsequently, a scratch was made using a 200-μL pipette tip, followed by incubation for 12 h in a starvation medium supplemented with 1% FBS. Scratch closure was observed under a microscope at 40× magnification and analyzed using ImageJ.

Trans-well invasion assay

A 24-well hanging cell culture insert (SPL Life Sciences, Gyeonggi-do, South Korea) was used. Each chamber was coated with Matrigel matrix diluted in serum-free RPMI-1640. SKOV3 cells were seeded in serum-free medium in the upper chamber coated with diluted Matrigel matrix. Complete medium was added to the bottom chamber for invasion. After 48 h, upper-chamber medium was aspirated and the upper chamber was washed with PBS. The cells were fixed with formaldehyde, permeabilized with 100% methanol, and stained with 0.5% crystal violet diluted in 20% methanol.

Tube-formation assay

First, 24-well plates were coated with Matrigel matrix (growth factor reduced, phenol-free; BD Bioscience, Franklin Lakes, NJ, USA) and allowed to solidify at 37 °C. SKOV3 cells treated with DHA/CBP or transfected with miR-200b were cultured in serum-free medium on Matrigel-coated plates. Likewise, HUVECs treated with SKOV3- or primary OC cell–conditioned medium (CM), VEGF-A, or DHA, or transfected with miR-200b and cultured in serum-free media on Matrigel-coated plates. Tube formation was observed using a microscope, and tube length was measured using ImageJ.

Cell viability assay

Cell viability was assessed using a WST-1 assay kit (Deaillab, Seoul, South Korea) according to the manufacturer's protocol. SKOV3 cells were seeded in 96-well plates at a density of 2 × 10^3 cells/well in complete medium and treated with DHA or CBP for 72 h. Subsequently, the cells were incubated with the

WST-1 reagent for 3 h at 37 °C. Absorbance was measured at 450 nm using a BioTek microplate reader (Winooski, VT, USA).

Blood biochemical analysis

On day 28 post cell injection, all surviving animals were anesthetized with ether and subjected to laparotomy. Blood samples were collected from the heart and sent to the Global Clinical Central Lab in Gyeonggi-do, Republic of Korea, for biochemical analyses. Immune response was assessed using white blood cell count, hemoglobin level, and platelet count. Liver function was evaluated by measuring the activities of various enzymes, including aspartate transaminase (AST), alkaline phosphatase (ALP), and alanine transaminase (ALT). Kidney function was assessed by measuring serum creatinine levels.

Correlation and Survival analysis using The Cancer Genome Atlas (TCGA-OV)

Kaplan–Meier survival analyses were performed by dichotomizing expression values into high and low groups based on the optimal cutoff values determined using the maximally selected rank statistic method. Overall survival was defined as the time from the date of initial diagnosis to the date of death or last follow-up. To reduce the influence of extremely long-term survivors, the overall survival duration was truncated at 3,000 days. Multivariate survival analysis was performed using the Cox proportional hazards model. All analyses were conducted using R (version 4.1.1).

Supplementary Figures and legends

a

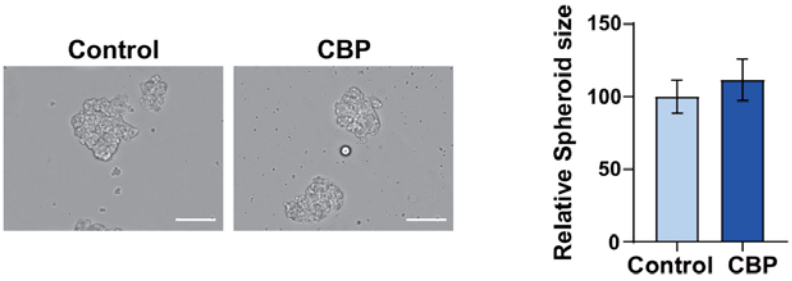

Supplementary Fig. 1. Effect of CBP on SKOV3 spheroid size. a Representative images of SKOV3 spheroids after treatment with 50 µM CBP. No significant changes were observed in spheroid size in response to treatment with 50 µM CBP. Scale bar: 100 µm.

a

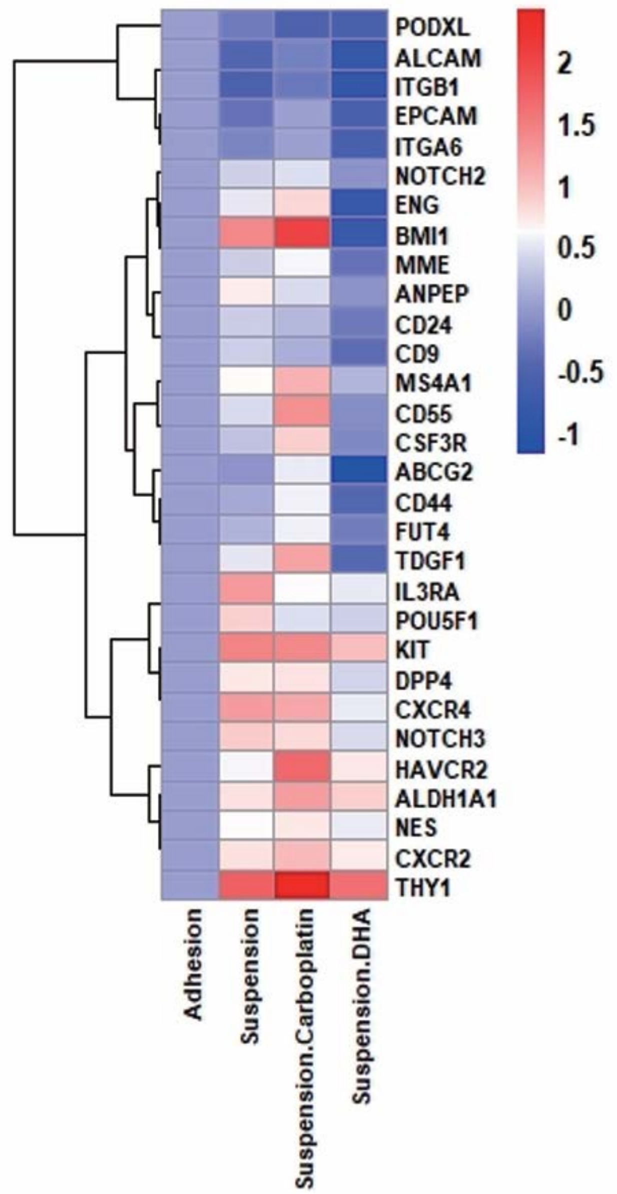

Supplementary Fig. 2. A human cancer stem cell marker qPCR array to analyze the anti-CSC effects of CBP and DHA. a DHA regulated the expression of CSC markers, especially BMI-1, which is upregulated in spheroids, whereas CBP treatment exerted little to no inhibitory effect.

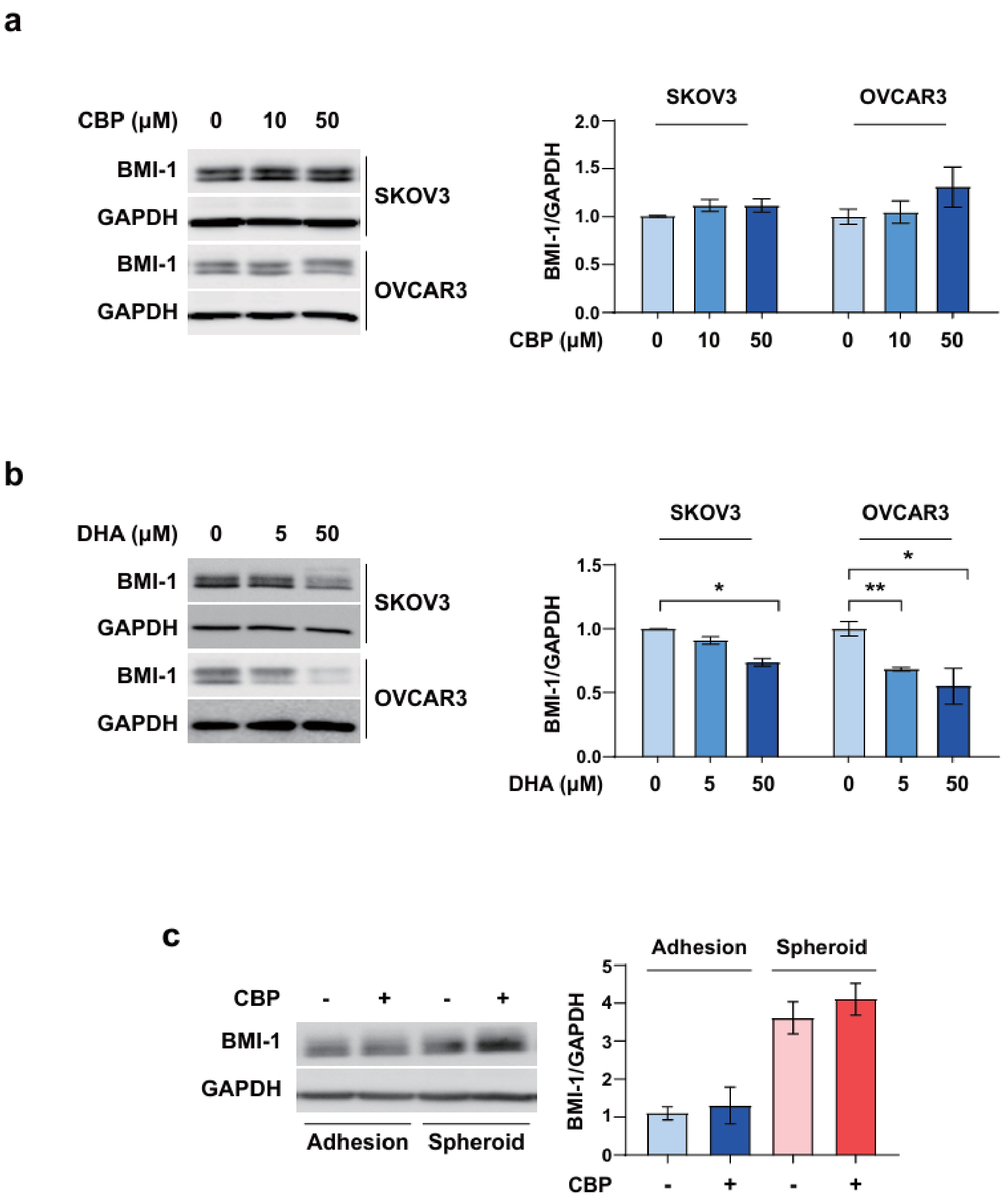

Supplementary Fig. 3. Effect of CBP and DHA on BMI-1 protein expression in SKOV3, OVCAR3, and primary ovarian cancer cells. a,b Western blot analysis of BMI-1 protein expression in adherent SKOV3 and OVCAR3 ovarian cancer cells treated with CBP and DHA. DHA treatment markedly reduced BMI-1 protein expression in both the cell lines. *$P < 0.05$, **$P < 0.01$ compared with controls by one-way analysis of variance (ANOVA) with Bonferroni's multiple comparison test. Error bars, standard error of the mean. **c** BMI-1 protein expression in adherent and spheroids SKOV3 cells. No significant changes were observed in protein expression after treatment with 50 μM CBP.

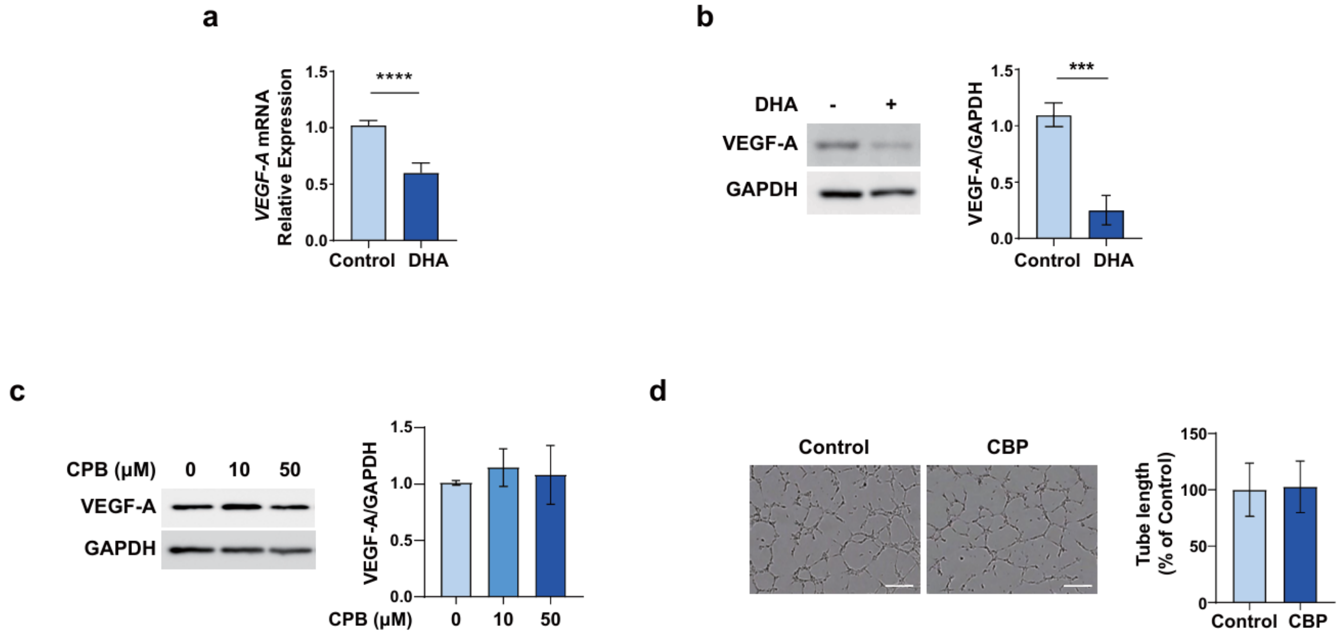

Supplementary Fig. 4. Effect of DHA and CBP on VEGF-A protein expression and tube formation in OVCAR3 and SKOV3. a,b VEGF-A mRNA and protein levels decreased in response to DHA in OVCAR3 cells. ***P < 0.001, ****P < 0.0001 for comparisons tested using unpaired two-tailed Student's t-tests. Error bars indicate standard error of the mean. **c** VEGF-A protein expression in SKOV3 cells. No significant changes were observed in protein expression in response to treatment with 50 μM CBP. **d** Conditioned media (CM) collected from CBP-treated SKOV3 cells had no significant effect on EC tube formation. Scale bar: 500 μm.

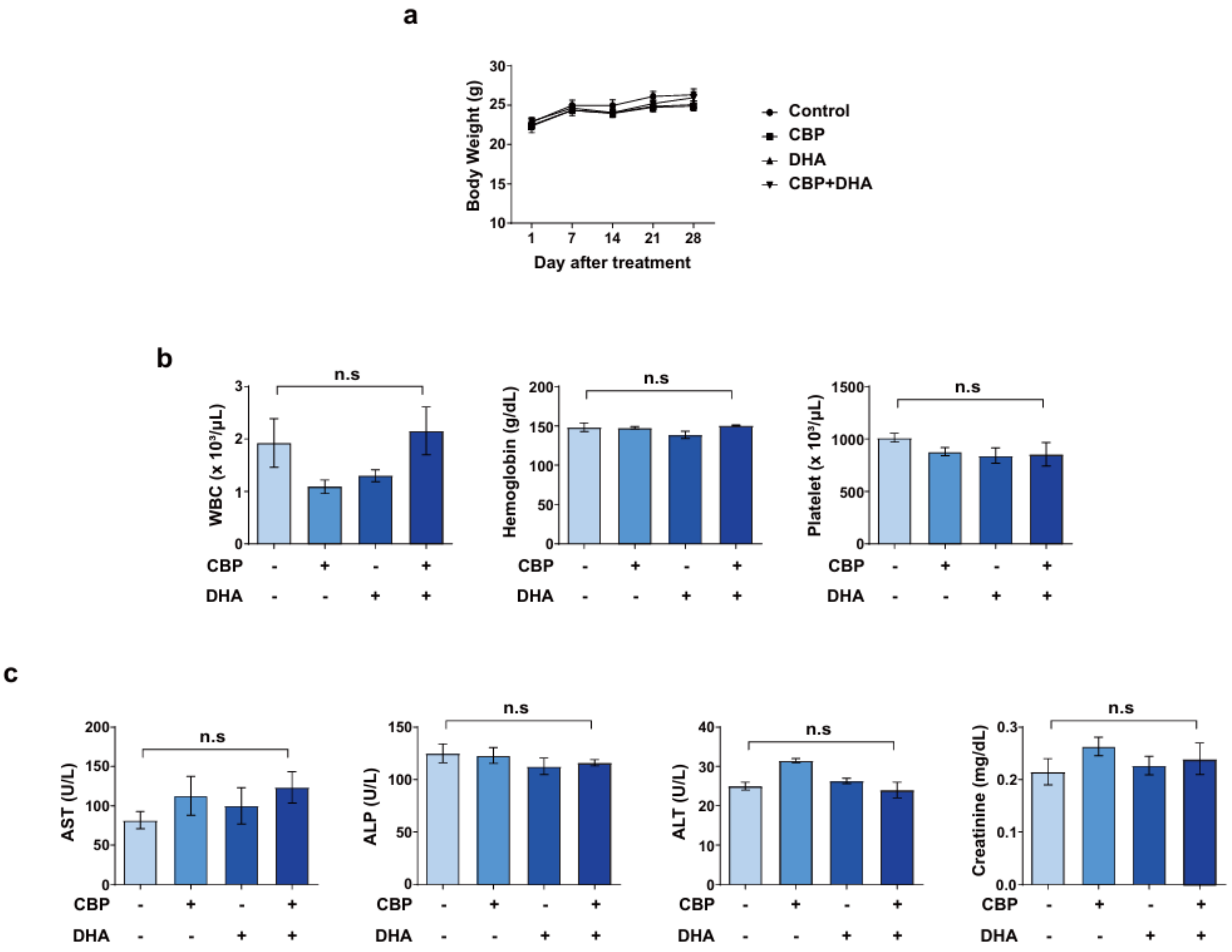

Supplementary Fig. 5. Effect of CBP and DHA on body weight and hematological parameters of SKOV3 xenograft mice. a Body weight trajectory of ovarian cancer-bearing mice treated with control (saline), CBP, DHA, or a combination of CBP and DHA over a 28-day period. Body weight remained stable across all treatment groups, suggesting that the treatments did not induce any detrimental effects on overall health. **b,c** Blood samples collected from the mice at the end of the treatment regimen were subjected to hematological assessment. The parameters analyzed included white blood cell (WBC) count, hemoglobin concentration, platelet count, aspartate aminotransferase (AST), alkaline phosphatase (ALP), and alanine aminotransferase (ALT) activity, and creatinine levels. Where indicated, intergroup comparisons did not reveal any statistically significant differences (ns).

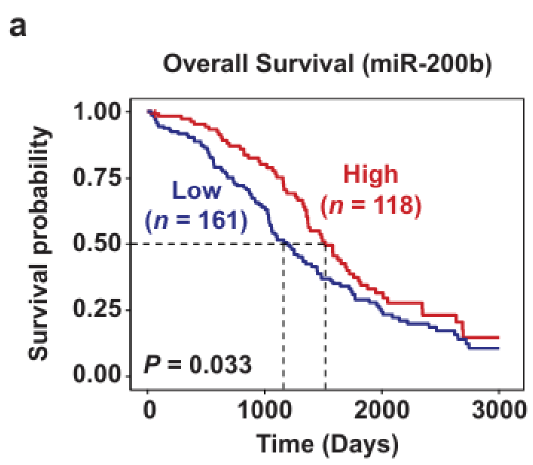

Supplementary Fig. 6. Survival analysis based on miR-200b and BMI-1 expression. a,b Kaplan–Meier plots comparing the overall survival between high- and low-expression groups for miR-200b and BMI-1, in TCGA ovarian cancer samples. P-values (P) from the log-rank test are shown.

Supplementary tables

Supplementary Table 1. Relationship between clinicopathologic characteristics and VEGF-A/BMI-1 expression in 107 OCs cases**.**

Variable	n (%)	VEGF-A			BMI-1		
		Negative, n (%)	Positive, n (%)	*P*	Negative, n (%)	Positive, n (%)	*P*
**Age (year)**				.690			.362
≤51[a]	52 (48.6)	16 (51.6)	36 (47.4)		40 (51.3)	12 (41.4)	
>51	55 (51.4)	15 (48.4)	40 (52.6)		38 (48.7)	17 (58.6)	
**FIGO stage**				.186			.298
I/II	40 (37.4)	15 (48.4)	25 (32.8)		30 (48.7)	10 (34.5)	
III/IV	67 (62.6)	16 (51.6)	51 (67.1)		48 (51.3)	19 (65.5)	
**Histology**				.682			.381
Serous	61 (57.0)	18 (58.1)	43 (56.6)		41 (52.6)	20 (69.0)	
Mucinous	16 (15.0)	3 (9.7)	13 (17.1)		14 (17.9)	2 (6.9)	
Endometrioid	7 (6.5)	3 (9.7)	4 (5.3)		5 (6.4)	2 (6.9)	
Clear cell	23 (21.5)	7 (22.6)	16 (21.1)		18 (23.1)	5 (17.2)	
**Histologic grade**				.277.			.385
1	25(23.4)	10 (32.3)	15 (19.7)		20 (25.6)	5 (17.2)	
2/3	82 (76.6)	21 (67.7)	61 (80.3)		58 (74.4)	24 (82.7)	
**CA-125 (Unit/mL)**							.866
≤646[b]	86 (80.4)	26 (83.9)	60 (78.9)	.789	63 (80.8)	23 (79.3)	
>646	21 (19.6)	5 (16.1)	16 (21.1)		15 (19.2)	6 (20.7)	
**Residual mass**				.388			.415
<1 cm	91 (85.0)	28 (90.3)	63 (82.9)		65 (83.3)	26 (89.7)	
≥1 cm	16 (15.0)	3 (9.7)	13 (17.1)		13 (16.7)	3 (10.3)	
**Adjuvant chemotherapy**				.566			.815
No	17 (15.9)	6 (19.4)	11 (14.5)		12 (15.4)	5 (17.2)	
Yes	90 (84.1)	25 (80.6)	65 (85.5)		66 (84.6)	24 (82.8)	

[a]Mean age; [b]Mean level; [c]Conventional platinum-based therapy.
FIGO, The International Federation of Gynecology and Obstetrics; CA-125, cancer antigen 125

Supplementary Table 2. Relationship between VEGF-A and BMI-1 status (determined using IHC) and progression-free survival in OC.

**Variable**	**Progression-free survival (months)**	
	**HR (95% CI)**	***P***
VEGF-A negative - BMI-1 negative	1	
VEGF-A positive - BMI-1 negative	2.158 (0.929–5.009)	.043
VEGF-A negative - BMI-1 positive	3.602 (1.460–7.096)	.036
VEGF-A positive - BMI-1 positive	3.863 (1.602–9.312)	.003

HR, hazard ratio; CI, confidential interval;
*Tested by COX proportional hazards model in which the reference is the VEGF-A positive - BMI-1 negative group, and adjusted with FIGO stage, histologic grade, CA-125 and residual mass.

Supplementary Table 3. Antibodies used for western blotting and immunohistochemistry.

**Antibodies**	**Source**	**Catalogue**	**Application**
BMI-1	Cell Signaling Technology	6964	western blot/ Immunohistochemistry
	Santa Cruz Biotechnology	SC-390443	Immunohistochemistry
VEGF-A	Abcam	ab1316	western blot/ Immunohistochemistry
VEGFR2	Cell Signaling Technology	2479	western blot
GAPDH	Cell Signaling Technology	2118	western blot

Supplementary Table 4. Sequence of primers used for qRT-PCR.

Gene	**Species**	**Forward Primer(5’-3’)**	**Reverse Primer (5’-3’)**
18S	Human	ACCCGTTGAACCCCATTCGTGA	GCCTCACTAAACCATCCAATCGG
BMI-1	Human	GACTCTGGGAGTGACAAGGC	ACTGGAGTACTGGGGCTAGG
VEGF-A	Human	TTGCCTGCTGCTCTACCTC	GATGGCAGTAGCTGCGCTGA
VEGFR2	Human	CTCTTGGCCGTGGTGCCTTTG	GTGTGTTGCTCCTTCTTTCAAC
